# Predictive Models of Tumour Response to Treatment Using Functional Imaging Techniques

**DOI:** 10.1155/2015/571351

**Published:** 2015-03-09

**Authors:** Loredana G. Marcu, Eva Bezak, Iuliana Toma-Dasu, Alexandru Dasu

**Affiliations:** ^1^Faculty of Science, University of Oradea, 410087 Oradea, Romania; ^2^School of Chemistry & Physics, University of Adelaide, Adelaide, SA 5000, Australia; ^3^Department of Physics, Royal Adelaide Hospital, Adelaide, SA 5000, Australia; ^4^Medical Radiation Physics Division, Stockholm University and Karolinska Institutet, 171 76 Stockholm, Sweden; ^5^Department of Medical and Health Sciences, Radiation Physics, Linköping University, 581 85 Linköping, Sweden

The aim of the current special issue was to bring together articles on various aspects of tumour modelling focusing on treatment response and prediction of clinical outcome based on functional imaging techniques.

Together with technological and radiobiological advances, tumour modelling is an emerging area of oncology, which plays a key role in predicting treatment outcome in cancer patients. Despite the complexity of tumour biology and its microenvironment, computational and mathematical models of virtual cancer behaviour and response to treatment are successfully developed and employed for clinical research. A mathematical (delay differential) model with optimal control for tumour-immune response with chemoimmunotherapy is presented by F. A. Rihan et al. in their research paper that describes the interaction of tumour cells and immune response cells with external therapy. In their model, the authors propose a numerical technique to solve the optimal control problem and identify the best strategy to combine chemotherapy and immunotherapy in order to minimise tumour load while maximising the strength of the immune system.

Current diagnostic and imaging methods provide in-depth metabolic information that can be employed for tumour modelling. Latest technical and molecular advances in the field of nuclear medicine offer new possibilities in functional imaging, overcoming some of the confines imposed by previous diagnostic techniques. Positron emission tomography (PET) is the most advanced technology designed to provide metabolic information of disease, treatment monitoring, and also evaluation of treatment outcome. Together with other quantitative imaging techniques, such as dynamic contrast-enhanced MRI, PET is a promising diagnostic tool assisting in patient stratification for specific therapies, evaluation of drug efficacy, assessment of chemo- and radiotherapy outcome, and prediction of survival.

This research idea is underlined in the paper by M. Jennings et al., which focuses on PET-specific parameters and radiotracers in theoretical tumour modelling. The work reviews the use of PET/CT information applied to* in silico* models of tumour growth, development, and behaviour during treatment. Tumour-related parameters such as cell proliferation, hypoxia, angiogenesis, and pH as well as PET/CT-specific biophysical parameters by means of SUV (standardized uptake value) and Hounsfield units are revisited in the context of computational modelling of complex processed involving tumour kinetics and treatment outcome.

While several PET radiotracers have been developed for clinical use to target specific tumour properties, FDG (fluorodeoxyglucose) continues to be the most commonly used radionuclide in functional imaging as it offers unique information for tumour detection, staging, target definition, and response monitoring during radio- and chemotherapy. In this special issue, J. Jeong and J. O. Deasy have modelled the relationship between FGD uptake and tumour radioresistance as a function of the tumour microenvironment. The mechanistic model has considered cellular status to be dictated by glucose and oxygen content, showing that cells in the intermediate stress state (that receive glucose but not oxygen) present an increased avidity of FDG when compared to well-oxygenated cells. The role of hypoxia and the current status of hypoxia imaging including PET-specific markers are discussed by L. G. Marcu et al. in their review paper written in the context of head and neck cancer. Hypoxia-specific PET markers have been implemented in several clinical trials to quantify hypoxic tumour subvolumes for dose painting and personalised treatment planning. Tracer pharmacokinetics and PET-derived functional parameters serve as important input data for* in silico* models that aim to simulate or interpret the acquired image. The paper discusses two main streams of PET tracer modelling: (1) models of specific tracer/oxygen dynamics with the aim of simulating PET images leading to results that are in close agreement with real PET images and (2) utilisation of PET data within a separate tumour model with the aim of creating a more specific model that is predictive of response to treatment. The authors have collated analytical compartment models of tracer pharmacokinetics, stochastic models of treatment response using probability distribution functions, and reports on model predictions within clinical radiotherapy planning systems of dose distribution. A pertinent conclusion of the review is that the role of computer models based on functional imaging techniques in understanding patient-specific tumour behaviour is effusively justified.

The challenge of hypoxia in cancer management is further analysed by M. M. Iglesias et al. in a research article looking at the multimodality functional imaging in radiotherapy planning and the relationship between dynamic contrast-enhanced (DCE) MRI, diffusion-weighted MRI, and FDG-PET in order to develop predictive individualised models of tumour response to radiotherapy in head and neck cancer patients. For an optimal biologically guided radiotherapy, to obtain relevant datasets on tumour hypoxia and cellular density, there is a need to understand the correlations and interactions between various functional imaging modalities. Parameters such as SUV (PET), Hounsfield units, dose, ADC (apparent diffusion coefficient) maps (MRI), and contrast exchange coefficients (DCE-MRI) have been recorded for each patient from the study and the relationship between parameters analysed. The authors concluded that the above functional parameters based on different image datasets are valuable in describing in a complex manner tumour oxygenation and vascularisation, cell density, and tumour malignity offering, therefore, treatment personalisation and optimisation.

A central motif of today's cancer management is personalised treatment planning and delivery. While this trend is featured in all papers of the current special issue, a more focused view upon treatment individualisation via multidimensional radiotherapy is presented in the work of I. Toma-Dasu and A. Dasu. According to the authors, the key approach to maximise the individualisation of treatment is by combining multiparameter information from imaging with predictive information from biopsies and molecular analyses and also by monitoring tumour response to treatment. The emphasis of the paper is on biologically adapted radiation therapy (BIOART), which is based on both pretreatment conditions and intrinsic responsiveness assessed using functional imaging techniques. The authors suggest that the BIOART concept should replace the simple dose painting approach for a more optimal management of radioresistant subvolumes within the BTV (biological target volume). Functional imaging is shown to have the potential to provide a paradigm shift in treatment planning and optimisation that extends beyond target definition.

*In silico* modelling in cancer research was and continues to be a very important tool of treatment simulation and optimisation, contributing to a more personalised medicine for an improved patient outcome. The use of computational models for treatment assessment and outcome prediction is fast growing and the power of* in silico* models as preclinical tools becomes acknowledged. Therefore, multidisciplinary research leading to predictive models of tumour response to treatment using functional imaging techniques needs to be encouraged to enable further developments in the diagnostic and treatment of cancer.

*Loredana G. Marcu* *Loredana G. Marcu* *Eva Bezak* *Eva Bezak* *Iuliana Toma-Dasu* *Iuliana Toma-Dasu* *Alexandru Dasu* *Alexandru Dasu*

